# Edaravone Protects Trophoblast Cells From Hypoxic Injury in Preeclampsia: Inhibition of the PI3K/AKT Pathway as a Promising Therapeutic Approach

**DOI:** 10.1002/iid3.70097

**Published:** 2024-12-11

**Authors:** Xin Liu, Jun Wan, Ming Wei, Yanan Tong, Zhaomin Yao

**Affiliations:** ^1^ Department of Nuclear Medicine General Hospital of Northern Theater Command Shenyang Liaoning China; ^2^ Department of Blood Transfusion The Fifth Affiliated Hospital of Zhengzhou University Zhengzhou Henan China; ^3^ Department of General Practice Department The Fifth Affiliated Hospital of Zhengzhou University Zhengzhou Henan China; ^4^ College of Medicine and Biological Information Engineering Northeastern University Shenyang Liaoning China

**Keywords:** edaravone (EDA), hypoxia, PI3K/AKT pathway, preeclampsia (PE), trophoblast cells

## Abstract

**Objective:**

Preeclampsia (PE) is a multifaceted medical condition that manifests during pregnancy, characterized by hypertension and damage to multiple organs. In PE, the placenta's impaired functionality leads to continuous hypoxia in placental tissues, which is considered the primary cause of the condition. Inhibition of hypoxia‐induced injury in trophoblast cells presents a potential therapeutic strategy for PE. Edaravone (EDA) is a potent antioxidant with proven efficacy against various diseases and injuries, yet its impact on PE requires further exploration.

**Methods:**

Placenta tissues from pregnant women, with or without PE, were collected, and levels of hypoxia‐inducible factor (HIF‐1α), P‐AKT, AKT, and PI3K proteins were analyzed using Western blotting. An in vitro anoxia model was established by treating the human trophoblast cell line HTR‐8/SVneo with cobalt chloride (CoCl_2_). Standard techniques were employed to measure proliferation, apoptosis, and reactive oxygen species (ROS) production rates in the anoxic cells, with and without EDA treatment.

**Results:**

HIF‐1α, P‐AKT, AKT, and PI3K protein levels were significantly elevated in the placenta of the PE group compared with the control group. EDA mitigated the CoCl_2_‐induced decrease in HTR‐8/SVneo cell viability and reduced apoptosis and ROS production. Furthermore, EDA counteracted the activation of the PI3K/AKT pathway in CoCl_2_‐treated trophoblasts.

**Conclusion:**

EDA protected trophoblasts against hypoxic injury by inhibiting the PI3K/AKT pathway, suggesting that it may serve as a promising therapeutic option for PE.

## Graphical abstract

The flowchart of the entire experiment.

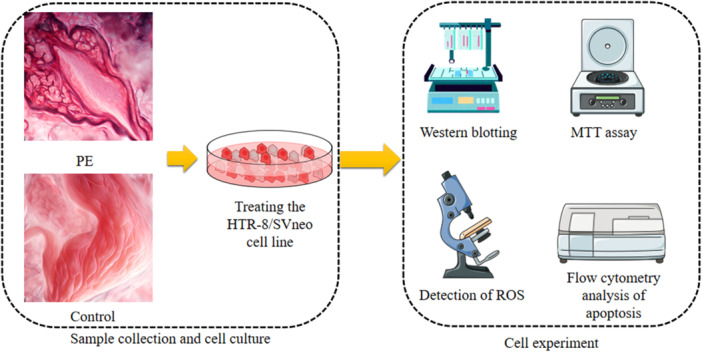

## 1 Introduction

Preeclampsia (PE) represents a multifaceted obstetric syndrome, typified by the emergence of hypertension and systemic organ dysfunction, which usually manifests subsequent to the 20th week of gestation. It is often associated with hepatic, renal, and cerebral perturbations [1, 2]. The placenta, serving as an indispensable organ for fetal respiration and nutrient uptake, is posited as a pivotal element in the underpinnings of PE [3, 4, 5]. Placental insufficiency, resulting from impaired placental perfusion, leads to chronic hypoxia in the placental environment. This condition is crucial in the development of PE. It is important to emphasize the connection between hypoxia and PE, as hypoxic conditions significantly elevate placental oxidative stress, promoting inflammation—a hallmark of pregnancies affected by PE [6, 7, 8]. The repercussions of PE on placental functionality can culminate in dire fetal developmental outcomes, evidenced by intrauterine growth restriction (IUGR) and reduced birth weights, which subsequently elevate the risk of neonatal mortality and predispose the offspring to lifelong health challenges [9]. The defining characteristic of the condition, a sustained hypoxic state within placental tissues, exacerbates these risks, thereby accentuating the critical need for efficacious management of PE to safeguard maternal health and the progeny's future well‐being [10, 11].

Addressing the hypoxia‐induced damage in trophoblast cells presents a viable avenue for the therapeutic intervention of PE, which is fundamentally associated with placental maladaptation [12, 13]. Trophoblasts, which are integral to the provision of nutrients and the facilitation of fetal development within the placenta, are particularly susceptible to oxygen deficit resulting from impaired blood flow, a salient factor in the pathogenesis of PE. By protecting these cells against hypoxic injury, it may be possible not only to preserve placental integrity but also to mitigate the clinical manifestations and avert the sequelae of PE. Hypoxic conditions in trophoblast cells trigger a harmful cascade that results in the release of factors causing vascular dysfunction both in the placenta and systemically. It is important to note that inflammatory conditions can also contribute to endothelial dysfunction. This highlights the complex mechanisms underlying the pathophysiology of PE [14]. Disrupting this deleterious progression could preclude the endothelial damage that is symptomatic of PE [15]. Furthermore, a prophylactic approach targeting placental hypoxia resonates with contemporary strategies in the management of PE, aiming to tackle the root cause rather than its downstream effects, with the potential to forestall the disease's onset or to attenuate its severity. Emerging molecular insights point to the possibility that enhancing the resilience of trophoblast cells to hypoxic conditions may alter gene expression patterns pertinent to placental development and function. This approach may correct the aberrant implantation and ontogenesis commonly observed in PE [16]. In sum, a profound understanding and intervention into the hypoxia‐induced impairment of trophoblast cells could be instrumental in formulating more efficacious treatments for this intricate condition.

While antioxidants have shown potential in ameliorating oxidative stress in trophoblast cells in vitro, their translation to clinical efficacy has been inconsistent. Edaravone (EDA), a potent antioxidant, has shown effectiveness in a variety of pathological contexts, including neurodegenerative disorders, cerebrovascular accidents, atherosclerotic disease, myocardial ischemia, and acute hepatic injury [17]. Its mode of action—through the attenuation of hydroxyl radicals and superoxide anions, the reduction of inflammation, and the mitigation of reperfusion injury—indicates prospective utility in the management of PE, although further research is warranted [18]. EDA's efficacy in counteracting hyperoxic injury, which occurs when cells are subjected to abnormally elevated oxygen levels resulting in the accumulation of reactive oxygen species (ROS), has been validated in rodent and human pulmonary epithelial cells, as well as in the preservation of retinal cells under oxidative duress [19]. Given that oxidative stress is a significant etiological factor in PE, the similarities in cellular injury across various cell types suggest that EDA's cytoprotective actions might extend to the trophoblasts affected by PE [20].

The current research seeks to explore the therapeutic potential of EDA on hypoxia‐induced cellular damage in the context of PE. Placental specimens from pregnant individuals with and without PE were scrutinized for the presence of hypoxia‐inducible factor (HIF‐1α), phosphorylated AKT (P‐AKT), AKT, and PI3K proteins utilizing Western blot analysis. Moreover, an in vitro model of anoxia employing the human trophoblast cell line HTR‐8/SVneo was subjected to cobalt chloride (CoCl_2_) exposure to emulate oxygen scarcity. This study assessed cellular proliferation, apoptotic rates, and ROS generation in anoxic conditions, with and without the administration of EDA, employing established biochemical assays. The findings from these investigations may shed light on EDA's effectiveness as a therapeutic agent for the oxidative stress component of PE, potentially paving the way for innovative treatment modalities.

## 2 Materials and Methods

### 2.1 Collection of Placental Tissue

This study recruited 20 patients diagnosed with PE and 20 control subjects exhibiting a normal pregnancy (absence of PE or other complications), all of whom underwent cesarean delivery at our institution. Inclusion in the study followed the acquisition of written informed consent and the endorsement of the Ethics Committee (ID: ky2021036). We collected sterilized placental tissues post‐delivery, conducting three washes with sterile saline. The tissue specimens were then segmented into cubic centimeter blocks and preserved in liquid nitrogen for subsequent analyses. The diagnosis of PE conformed to the guidelines stipulated by the International Society for Pregnancy and Hypertension. The clinical characteristics of the study subjects are presented in Table 1.

**Table 1:** Clinical data of patients.

Clinical Data	Control group	PE group	*p* values
Maternal age (year)	30.96 ± 3.35	32.73 ± 4.80	0.004
Maternal weight (kg)	74.41 ± 12.59	78.41 ± 10.44	NS
Parity	2.16 ± 1.05	2.86 ± 1.45	NS
Gestational age (week)	38.2 ± 1.98	34.1 ± 3.35	0.013
Systolic pressure (mm Hg)	112.46 ± 7.19	161.46 ± 18.84	0.00
Diastolic blood pressure (mm Hg)	69.50 ± 7.20	103.6 ± 14.85	0.015
Proteinuria (g/day)	0	3.86 ± 3.86	0.00
Cesarean section (n)	20	20	—
Neonatal birth weight (g)	3381.00 ± 480.64	2336.51 ± 823.14	0.014

*Note:* Data are presented as mean ± SD.

### 2.2 Trophoblast Cell Culture and Treatment

We procured the human trophoblast HTR‐8/SVneo cell line from the American Type Culture Collection (Manassas, VA, USA) and cultivated it in DMEM (HyClone; GE Healthcare Life Sciences, Logan, UT, USA), fortified with 10% fetal bovine serum (FBS; HyClone; GE Healthcare Life Sciences). This occurred within a humidified incubator (BINDER CB, China) under a 5% CO_2_ atmosphere at 37°C. Cells were cultured until reaching 80% to 90% confluency before passaging, with the growth medium refreshed every 3 days. Post 24‐h serum starvation, cells were rinsed with serum‐free DMEM and resowed in 6‐ or 96‐well plates. Hypoxia was induced using 500 μM CoCl_2_ (Z693847, Sigma, Germany), following which the cells received EDA (Mitsubishi Pharmaceuticals Corporation, Tokyo, Japan) treatment (100 μM).

### 2.3 MTT Assay

Cells were planted in a 96‐well plate at a density of 5 × 104 cells per well and cultured for 24 h. Subsequent treatment involved various concentrations of CoCl_2_ and EDA. After 24 h, 20 µL MTT (475989, Sigma, Germany) was introduced to each well, followed by an additional 4‐h incubation period. The medium was then replaced with 150 µL DMSO (X11263, Xiaoyou Biotechnology, China) to dissolve formazan crystals, and optical density (OD) at 570 nm was assessed using a Bio‐Tek ELX800 microplate reader (BioTek technology, America). Viability was determined by the equation OD_experiment_/OD_control_ × 100%. This process was replicated three times.

### 2.4 ROS Detection

Cells were co‐administered with 100 µM EDA and 500 μM CoCl_2_ for a period of 24 h. After 20‐min incubation with 10 µl DCFH‐DA (4091‐99‐0, BIOFOUNT technology, China) at 37°C, cells were rinsed twice with PBS (PRICELLA Biotechnology, China). Cells were then observed under a fluorescence microscope (OPTIKA IM‐5FLD, Italy), and the green fluorescence intensity of oxidized 2,7‐dichlorofluorescein (DCF) was analyzed using Image J software.

### 2.5 Flow Cytometric Analysis of Apoptosis

HTR‐8/SVneo cells, cultivated in 6‐well plates, underwent treatment with CoCl_2_ and/or EDA for 24 h. Post trypsinization (PRICELLA Biotechnology, China) and PBS washing, cells were resuspended in the Annexin‐V‐Fluos binding buffer supplied in the apoptosis staining kit (BD Bioscience) and stained with annexin‐V‐Fluos and propidium iodide (PI) (BD Bioscience) for 20 min at room temperature. The stained cells were processed through a flow cytometer to analyze the percentage of apoptotic cells. This experiment was conducted three times.

### 2.6 Western Blotting

Placental tissues and treated trophoblasts were homogenized in RIPA lysis buffer (Sigma, Germany), inclusive of a phosphatase inhibitor at a 10:1 ratio. The protein concentration of the lysates was determined using a BCA Protein Concentration Assay Kit (Beyotime Biotechnology, Haimen, China). Subsequently, 20 μg of proteins per sample were isolated through SDS‐PAGE (Shanghai Yongke Biotechnology Co., LTD, China) and transferred to polyvinylidene fluoride (PVDF) membranes (Shanghai Yongke Biotechnology Co., LTD, China). Following a 1‐h blocking phase with 5% skim milk in PBST, the membranes underwent overnight incubation with primary antibodies at 4°C. These antibodies targeted β‐actin (Abcam, MA, ab8227), PI3K (ab191606, Abcam, MA), P‐AKT (ab38449, Abcam, MA), AKT (sc‐5298, Santa Cruz Biotechnologies, Dallas, TX), HIF‐1α (sc‐8711, Santa Cruz Biotechnologies, Dallas, TX), Bax (sc‐7480, Santa Cruz Biotechnologies, Dallas, TX), and Bcl‐2 (sc‐7382, Santa Cruz Biotechnologies, Dallas, TX). WB special antibody diluents are purchased from Absin company (Shanghai, China, product code: abs954, for detection antibody and conjugated antibody). The blots were washed thrice with TBST and then incubated with HRP‐linked secondary antibody for 2 h at room temperature. Protein bands were visualized using enhanced chemiluminescence reagents and quantified using Image J software. This experiment was conducted three times.

### 2.7 Statistical Analysis

Statistical analyses were conducted using SPSS software version 21.0. All data are expressed as the mean ± standard deviation from three independent experiments. The independent sample student's *t*‐test was employed for comparing two groups, while one‐way ANOVA with a Tukey post‐hoc test was utilized for multiple comparisons. A *p*‐value of less than 0.05 was deemed statistically significant.

## 3 Results

### 3.1 Activation of the PI3K/AKT Signaling Pathway in PE Placenta

**Figure 1:**
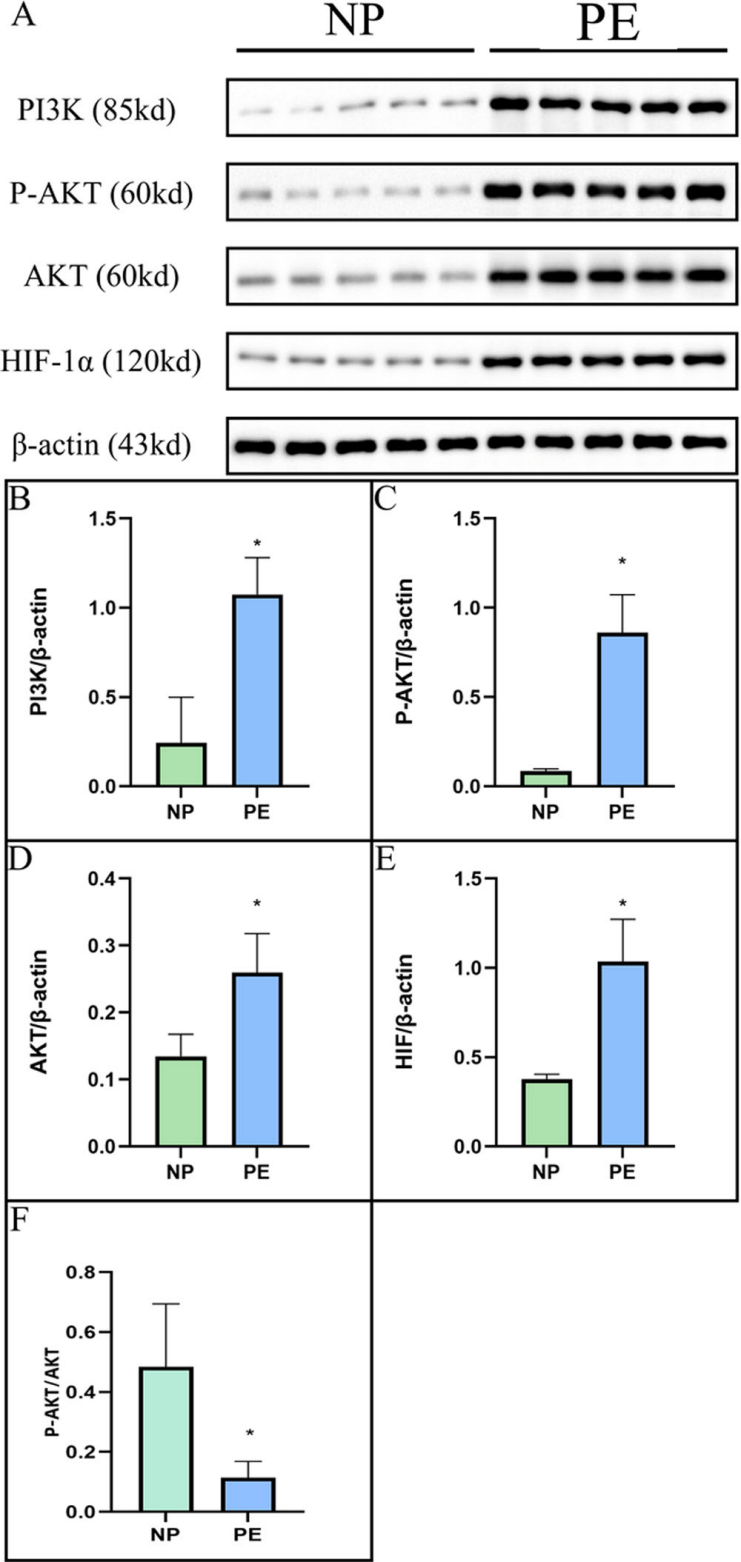
Immunoblot showing placental HIF‐1α, AKT, P‐AKT, and PI3K levels in normal (*n* = 20) and PE patients (*n* = 20). A. The Western blot figures of HIF‐1α, AKT, P‐AKT, and PI3K. B–E. The histogram of PI3K, P‐AKT, AKT, and HIF expression, respectively. F. Comparison between P‐AKT and total AKT (*p < 0.05 compared with the control group. NP, normal patient; PE, preeclampsia patient). All samples were run in triplicate.

Considering the crucial role of the PI3K/AKT signaling pathway in the progression of PE, we assessed the expression levels of PI3K, P‐AKT, AKT, and HIF‐1α proteins in placental tissues extracted from healthy subjects and PE patients. As depicted in Figure 1, there was a significant elevation in the expression of all these proteins in preeclamptic tissues when compared with the controls.

### 3.2 EDA Counteracts CoCl_2_‐Induced Hypoxic Injury in HTR‐8/SVneo Cells

**Figure 2:**
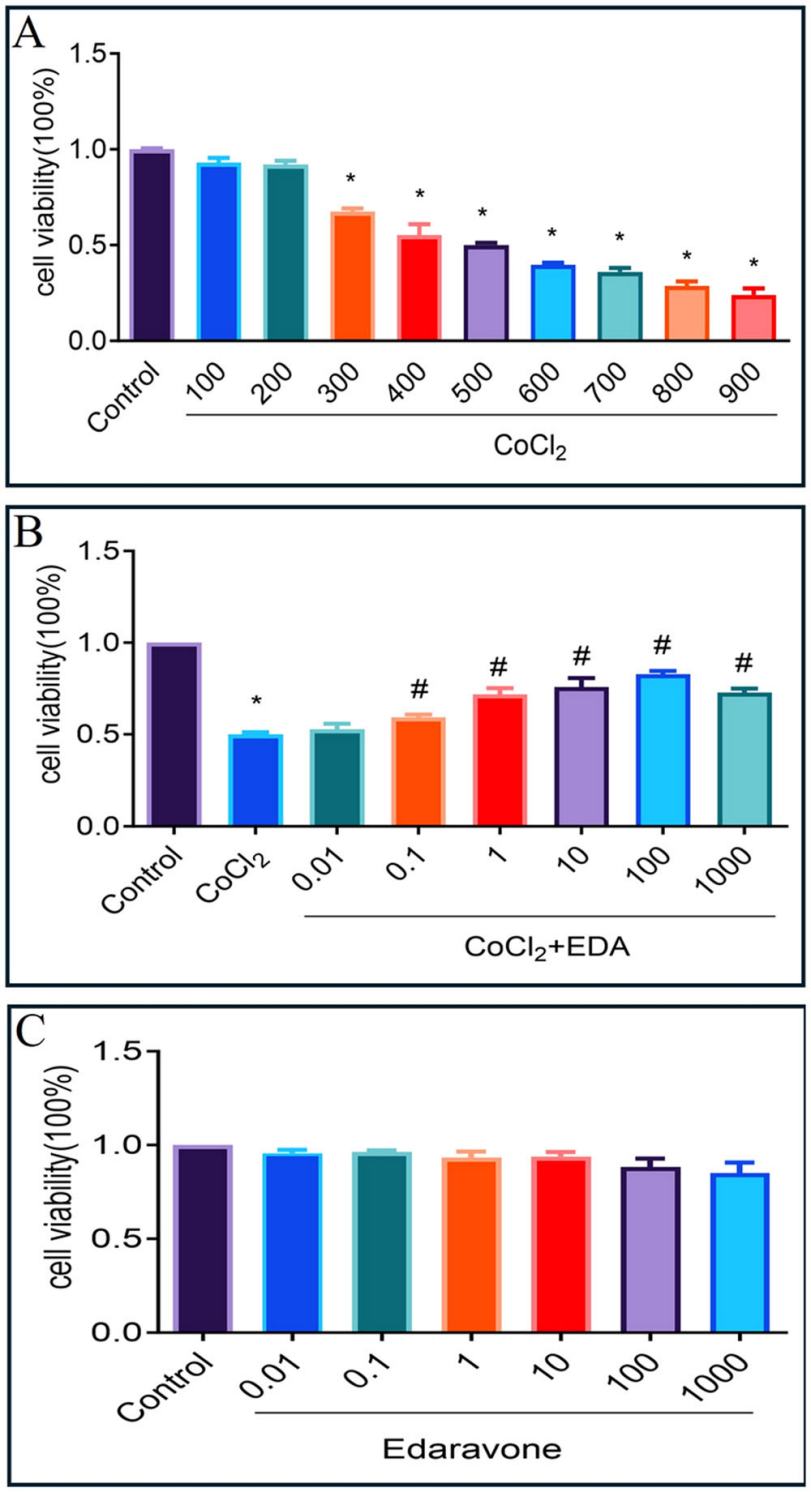
Viability of trophoblasts treated with EDA and CoCl_2_. A. The cell viability of H8 cells with different concentrations of CoCl_2._ B. The cell viability of H8 cells with mixing of 500 μM CoCl_2_ and different concentrations of EDA. C. The cell viability of H8 cells with different concentrations of EDA. (*p < 0.05 compared with the control group; ^#^ *p* < 0.05 compared with the CoCl_2_ treatment group. EDA, edaravone.) All experiments were repeated three times.

Figure 2A illustrates that the viability of HTR‐8/SVneo cells diminishes in a concentration‐dependent manner when exposed to CoCl_2_ within a range of 100 to 900 μM. In contrast, Figure 2C indicates that EDA, when administered across a broad concentration spectrum from 0.01 to 1000 μM, does not significantly impact cell viability. For the experiments depicted in Figure 2B, a concentration of 500 μM CoCl_2_ was selected for coadministration with EDA at varying concentrations (0.01–1000 μM). Alongside, a control sample and a sample containing exclusively 500 μM CoCl_2_ were analyzed for comparison. Notably, the co‐treatment with 100 μM EDA was found to effectively mitigate the CoCl_2_‐induced decline in cell viability.

**Figure 3:**
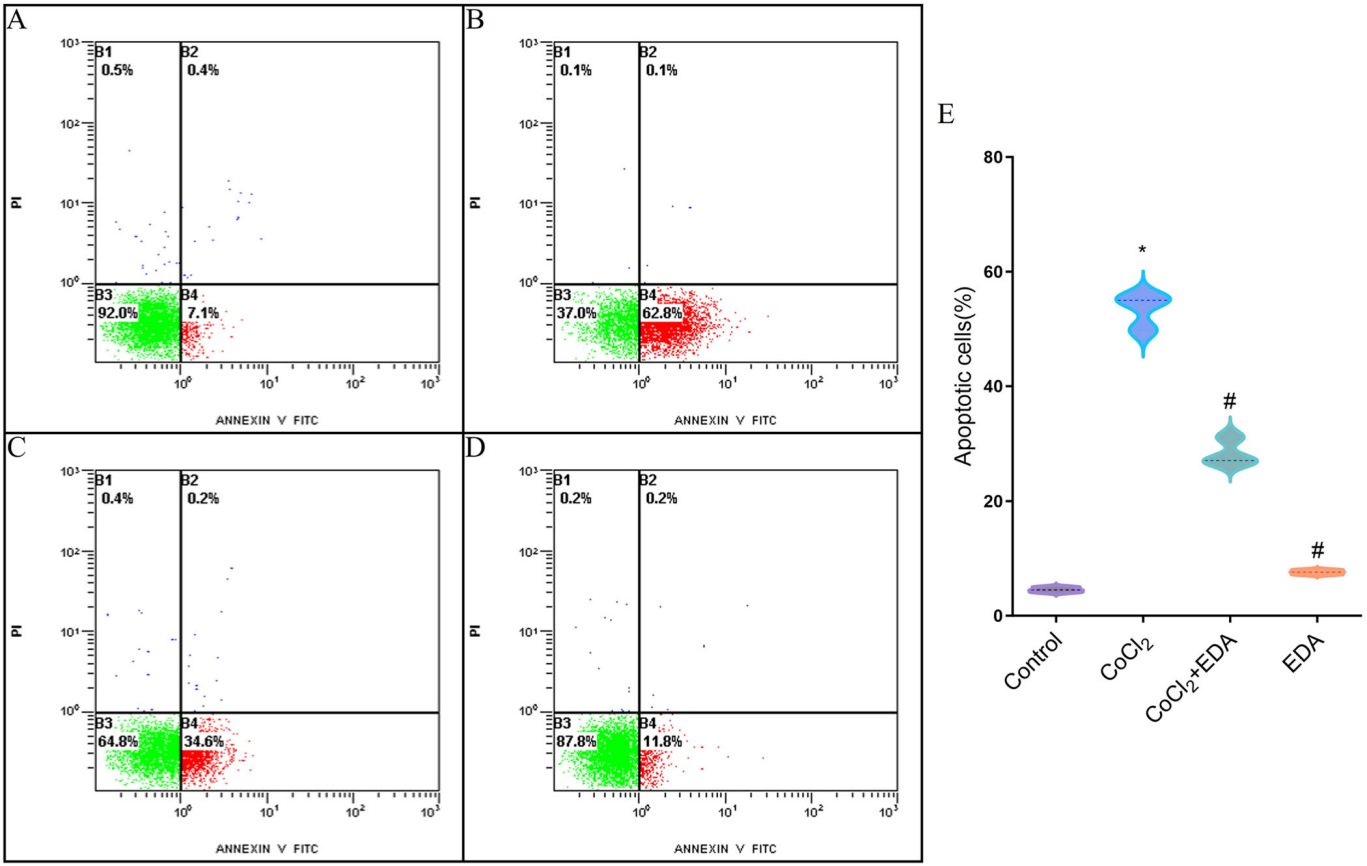
The protective effect of EDA against CoCl_2_‐induced apoptosis. A. Cell apoptosis distribution of H8 cells in control group. B–D. Illustrates graphical representation of flow cytometry data for cell apoptosis distribution of H8 cells exposed to CoCl_2_, CoCl_2_ + EDA, and EDA groups, respectively. E. The histogram of cell apoptosis in different groups. (*p < 0.05 compared with the control group; ^#^ *p* < 0.05 compared with the CoCl_2_ treatment group. EDA, edaravone.) All samples were run in triplicate.

**Figure 4:**
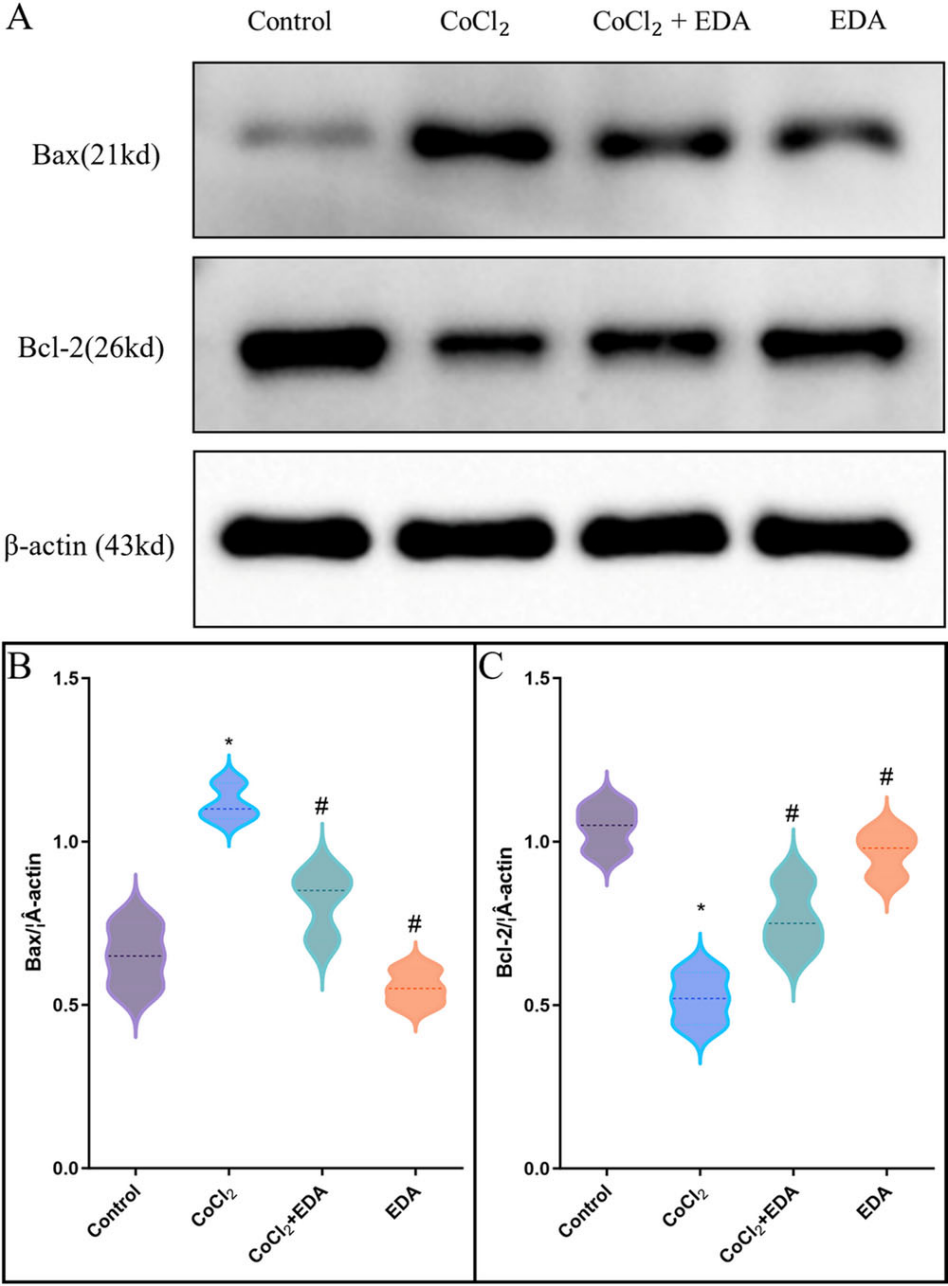
Immunoblot showing Bcl‐2 and Bax protein expression in cells treated with CoCl_2_ and EDA. A. The Western blot figures of Bcl‐2 and Bax. B. The histogram of Bax expression. C. The histogram of Bcl‐2 expression. (*p < 0.05 compared with the control group; ^#^ *p* < 0.05 compared with the CoCl2 treatment group. EDA, edaravone.) All experiments were repeated three times.

As evidenced in Figure 3, the percentage of apoptotic cells increased markedly in the presence of CoCl_2_ (55.3% ± 1.65%) compared to the untreated cells, a trend that was mitigated by pretreatment with EDA (*p* < 0.05). In line with these findings, CoCl_2_ upregulated the proapoptotic protein Bax and downregulated the antiapoptotic Bcl‐2 in trophoblasts, with both levels being normalized by EDA (Figure 4). These findings collectively imply that EDA effectively safeguards trophoblasts from the deleterious effects of CoCl_2_‐induced hypoxia.

### 3.3 Reduction of Hypoxia‐Induced ROS Production in Trophoblasts by EDA

**Figure 5:**
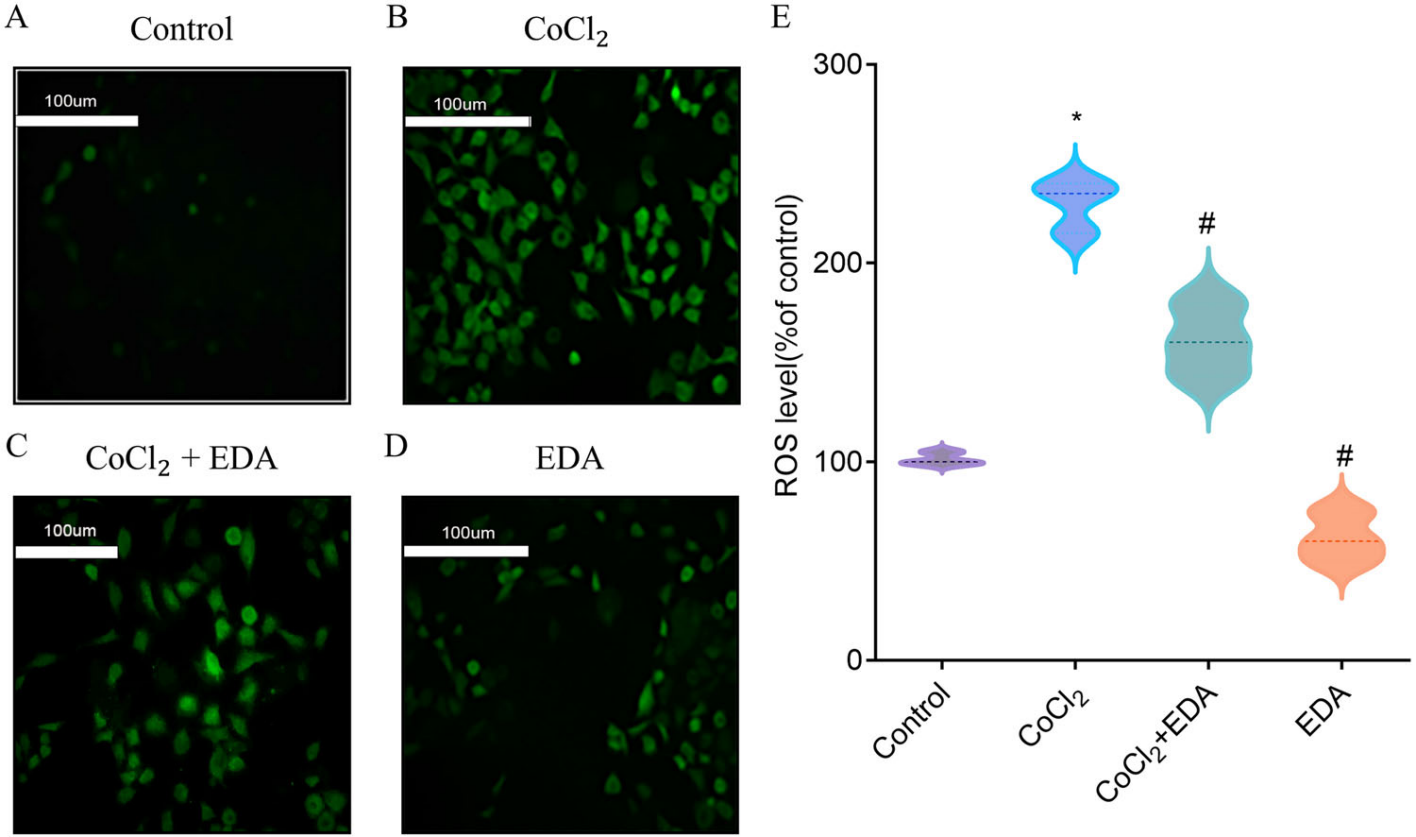
The effect of EDA on ROS levels in the different groups. A–D. The fluorescence figure of H8 cells in Control, CoCl_2_, CoCl_2_ + EDA, and EDA groups. E. The histogram of ROS level of different groups. (*p < 0.05 compared with the control group; ^#^ *p* < 0.05 compared with the CoCl_2_ treatment group. EDA, edaravone.) All samples were run in triplicate.

We assessed the level of intracellular ROS generated by CoCl_2_ using the DCFH‐DA probe. As shown in Figure 5, cells treated with 500 μM CoCl_2_ exhibited a substantial increase in DCF fluorescence intensity compared to untreated controls. However, the presence of EDA significantly curtailed the ROS levels in hypoxic cells.

### 3.4 EDA Inhibits CoCl_2_‐Induced Activation of the PI3K/AKT Pathway

**Figure 6:**
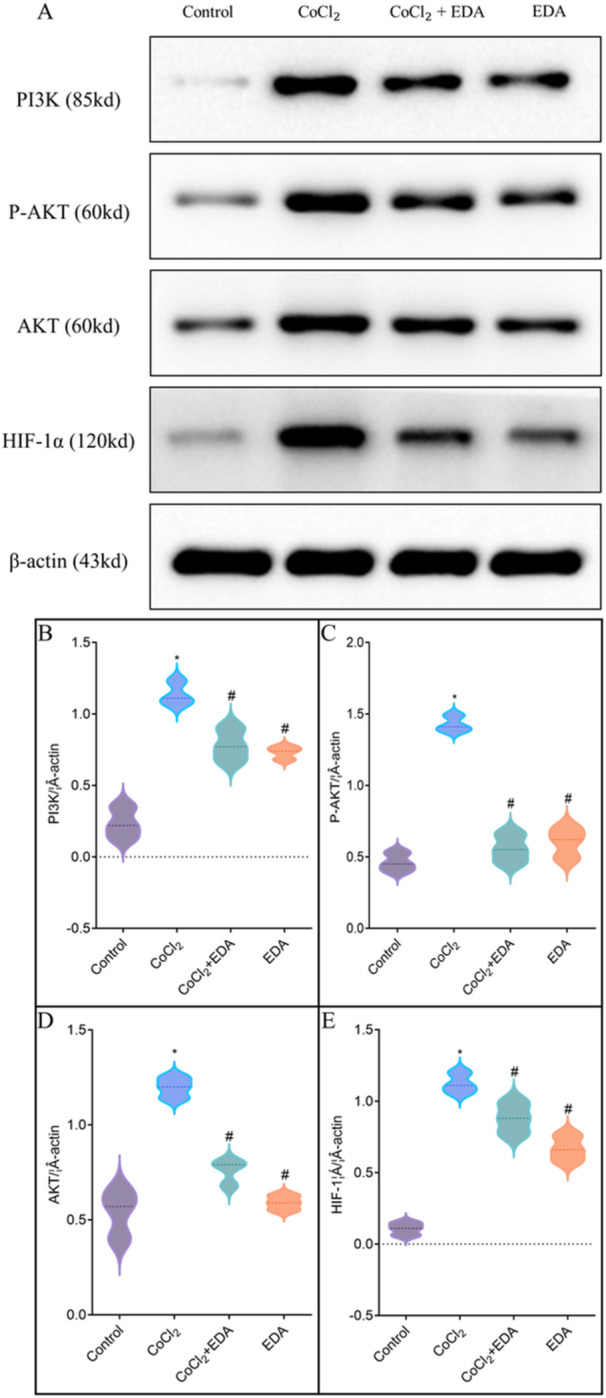
PI3K, P‐AKT, AKT, and HIF‐1α protein expression in the cells treated with CoCl_2_ and EDA. A. The Western blot figures of PI3K, P‐AKT, AKT, and HIF‐1α. B–E. The histogram of PI3K, P‐AKT, AKT, and HIF expression, respectively. (*p < 0.05 compared with the control group; ^#^ *p* < 0.05 compared with the CoCl2 treatment group. EDA, edaravone.) All experiments were repeated three times.

Previous research indicates that hypoxia‐induced oxidative stress may trigger apoptosis via the activation of the PI3K/AKT pathway. HTR‐8/SVneo cells treated with CoCl_2_ for 24 h expressed notably higher levels of p‐AKT, AKT, and PI3K compared with untreated controls (Figure 6). The additional administration of EDA normalized the expression levels of all these proteins. Therefore, EDA confers protection to HTR‐8/SVneo cells against CoCl_2_‐induced oxidative stress by inhibiting the PI3K/AKT pathway.

## 4 Discussion

Our study elucidates the protective effects of EDA on trophoblast cells challenged by hypoxic conditions, a scenario that mirrors the pathophysiological conditions prevalent in PE. We have demonstrated that EDA effectively counters the adverse impacts of hypoxia, induced by cobalt chloride (CoCl₂) treatment, in the HTR‐8/SVneo cell line. This cell line is a recognized in vitro model for examining trophoblast function, making our findings particularly relevant.

In Table 1, we compared the basic clinical information of the patients, with gestational age emerging as a particularly notable indicator. While our findings showed statistical significance, it is important to acknowledge that PE is a significant contributor to severe adverse perinatal outcomes and iatrogenic premature delivery. Many pregnant women with PE are forced to terminate their pregnancies early due to serious complications, with 36.3% of iatrogenic preterm births linked to severe PE [21, 22]. Given these factors, the significance of our comparison of gestational age warrants further exploration. However, we recognize that our research has certain limitations, and we plan to investigate this issue more thoroughly in future studies. We observed a pronounced increase in HIF‐1α, P‐AKT, AKT, and PI3K proteins in placental tissues from PE patients compared to healthy controls. This upregulation underscores the critical role of the PI3K/AKT signaling pathway in the development of PE. This pathway, integral to cellular survival and proliferation, is known to be disrupted in various diseases, including cancer and inflammatory disorders. Within the context of PE, the activation of this pathway likely represents an adaptive mechanism to chronic hypoxia that, if unregulated, can lead to extensive cellular damage and the systemic manifestations of the disease.

Treatment with EDA resulted in a significant reduction in P‐AKT, AKT, and PI3K protein levels in CoCl₂‐exposed trophoblasts, indicating that EDA's protective mechanism involves modulation of this pivotal signaling pathway. Given the central role of the PI3K/AKT pathway in managing cellular responses to oxidative stress and survival under hypoxic conditions, EDA's inhibition of this pathway not only mitigates immediate hypoxic injury but also may alleviate the inflammatory and vascular complications associated with PE. Furthermore, our research recorded a decrease in apoptosis and ROS production in trophoblast cells treated with EDA, highlighting its antioxidant capabilities in curtailing oxidative stress—a major factor in cellular dysfunction and death in hypoxic environments. Reducing ROS levels is vital in preventing oxidative damage to cellular components such as lipids, proteins, and DNA, which perpetuates a cycle of injury and dysfunction in the placenta.

The clinical implications of these findings are profound. Demonstrating EDA's efficacy in a controlled in vitro environment sets a foundational stage for potential clinical trials aimed at exploring EDA as a therapeutic agent in PE management. Should similar protective effects be replicated in vivo, EDA could significantly enhance the current treatment landscape for PE, which predominantly focuses on symptom management rather than tackling the disease's root causes.

Importantly, the protective effect of EDA on trophoblast cells has broader implications for managing PE. By maintaining cell viability and function during hypoxic conditions, EDA may enhance placental perfusion and subsequently improve fetal outcomes. This is critical considering the established link between placental dysfunction and adverse neonatal outcomes, such as IUGR and preterm birth [23, 24]. Therefore, interventions that bolster trophoblast resilience could directly lead to improved pregnancy outcomes. However, while our results are promising, the translation of these findings into clinical practice necessitates careful consideration. PE's pathophysiology is multifaceted, involving numerous pathways and systemic effects beyond the placenta [25, 26, 27, 28]. Thus, while EDA shows potential in vitro, its in vivo effectiveness remains to be rigorously tested through clinical trials to determine optimal dosages, safety, and long‐term effects. Additionally, the timing of therapeutic intervention is crucial. Administering EDA early, before severe hypoxia sets in, might be essential for maximizing its therapeutic benefits. Such a preemptive strategy could potentially check the progression of PE and reduce the necessity for interventions like early delivery, which pose risks to both mother and child [29, 30].

In summary, our research supports the hypothesis that EDA protects trophoblast cells from hypoxic injury by inhibiting the PI3K/AKT pathway and reducing oxidative stress. These findings not only deepen our understanding of the molecular mechanisms at play in PE but also suggest a promising therapeutic route that could improve outcomes for mothers and their babies in PE‐afflicted pregnancies. Further studies are imperative to validate these results in clinical settings and to fully explore EDA's potential in the comprehensive management of PE.

## 5 Conclusion

PE, a complex pregnancy disorder characterized by hypertension and proteinuria, has been associated with oxidative stress and mitochondrial dysfunction. The primary focus of this investigation was to assess the role of EDA, a free radical scavenger, in mitigating the effects of hypoxia‐induced toxicity in an in vitro model of placental hypoxia. The findings underscored the significance of the PI3K/AKT pathway in PE, as well as the potential of EDA in reversing CoCl_2_‐induced cell damage. EDA demonstrated its capacity to counteract the toxic effects of hypoxia on human trophoblasts, primarily through its antioxidative and antiapoptotic properties. Moreover, EDA inhibited the CoCl_2_‐induced activation of the PI3K/AKT pathway, potentially offering a novel therapeutic target for managing PE.

Despite these promising findings, the study was not without limitations, including a small sample size and a lack of placenta histology and authentication. Further studies involving larger cohorts and more comprehensive experimental designs are warranted to validate these findings and to explore the precise mechanism by which EDA inhibits the PI3K/AKT pathway. Additionally, the potential therapeutic benefits of combining EDA with specific PI3K/AKT inhibitors merit further exploration.

## Author Contributions

**Xin Liu:** funding acquisition, writing–original draft. **Jun Wan:** data curation. **Ming Wei:** investigation. **Yanan Tong:** conceptualization. **Zhaomin Yao:** writing–review and editing.

## Ethics Statement

The research was approved by the ethical review committee of the Fifth Affiliated Hospital of Zhengzhou University (ID: ky2021036).

## Consent

The patient's consent for personal information and placenta collection was obtained.

## Conflicts of Interest

The authors declare no conflicts of interest.

## Supporting information

Supporting information.

## Data Availability

The analyzed data sets generated during the present study are available from the corresponding author upon reasonable request.
