# Supplementary figures and images for: Edaravone Protects Trophoblast Cells From Hypoxic Injury in Preeclampsia: Inhibition of the PI3K/AKT Pathway as a Promising Therapeutic Approach
Source: Immun Inflamm Dis. 2024 Dec 11;12(12):e70097. doi: 10.1002/iid3.70097 (PMC11633048; doi:10.1002/iid3.70097)

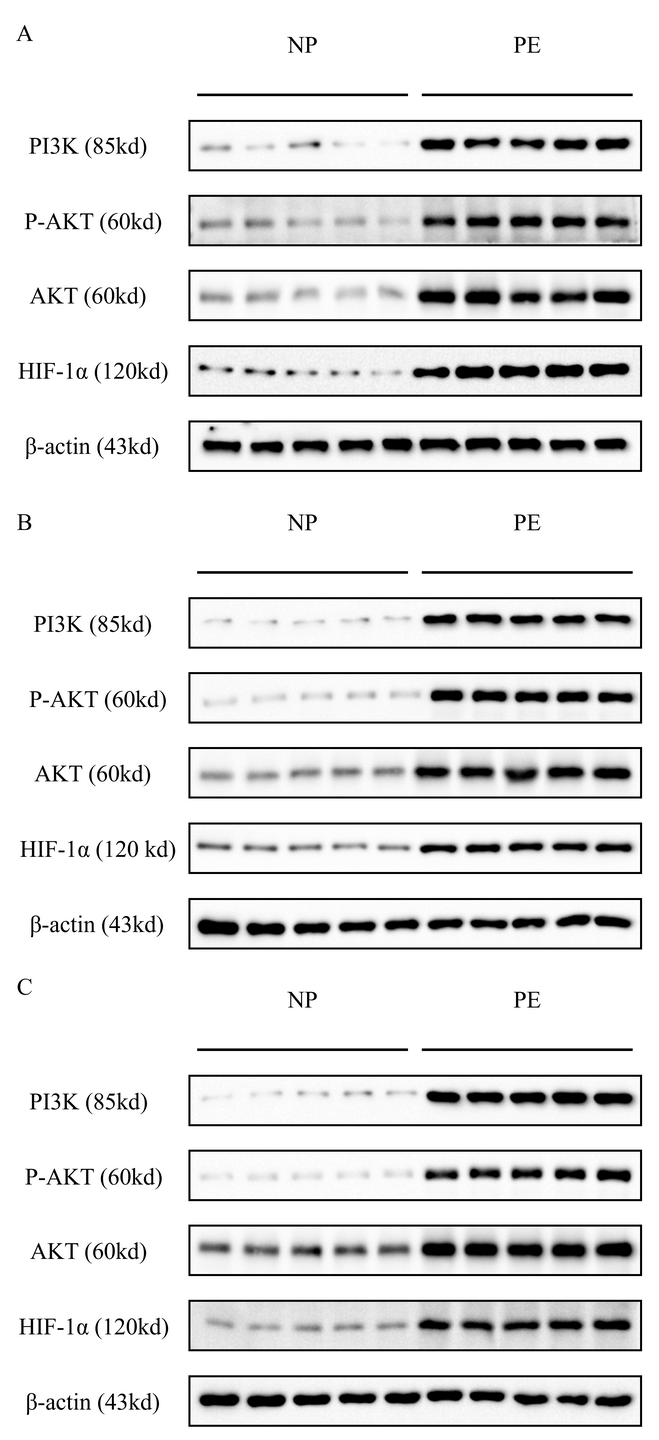

Supplement: Supplementary file 1 — Supporting information. [file IID3-12-e70097-s001.tif]
